# Magnetic-Immuno-Loop-Mediated Isothermal Amplification Based on DNA Encapsulating Liposome for the Ultrasensitive Detection of P-glycoprotein

**DOI:** 10.1038/s41598-017-10133-3

**Published:** 2017-08-24

**Authors:** Hongmei Cao, Xueen Fang, Peng Liu, Hua Li, Weiwei Chen, Baohong Liu, Jilie Kong

**Affiliations:** 1 Department of Chemistry and Institute of Biomedical Sciences, Fudan University, Shanghai, 200433 P.R. China; 2 Department of Laboratory Diagnosis, Changhai Hospital, Second Military Medical University, 168 Changhai road, Shanghai, 200433 China

## Abstract

Determination of proteins, especially low-abundance proteins with high sensitivity and specificity, is essential for characterizing proteomes and studying their biochemical functions. Herein, a novel Magnetic-Immuno-Loop-Mediated Isothermal Amplification (Im-LAMP) based on DNA-encapsulating liposomes (liposome-Im- LAMP), was developed for trace amounts of proteins. To the best of our knowledge, this is our first report about the magnetic Im-LAMP approach based on liposomes encapsulated template DNA as the detection reagent. The DNA template was released from liposomes and then initiated an Im-LAMP reaction, generating the fluorescence signal with high sensitivity and rapidity. This technique was applied for the determination of P-glycoprotein as a model protein. It was demonstrated that the technique exhibited a dynamic response to P-glycoprotein ranging from 1.6*10^−2^ to 160 pg/ml with a greatly low detection limit of 5*10^−3^ pg/ml (5 fg/ml) which is substantially better than conventional enzyme-linked immunosorbent assays (ELISA). This ultra sensitivity was attributed to the LAMP reaction initiated by the enormous DNA targets encapsulated in liposomes. This magnetic liposome-Im–LAMP as an alternative approach is attractive for applications in other low-abundance proteins detection in clinical diagnostics.

## Introduction

Nowadays, the detection and quantification of low-abundance proteins, in particular, the trace amounts of biomarkers in blood, is essential for indicating disease states and outcomes in clinical cancer treatment. Thus, developing a rapid and sensitive technique to detect low-abundance proteins has aroused wide research interest in recent years. Particularly, the development of amplification strategies to fabricate ultrasensitive protein detection has received great attention. In general, signal amplification strategies are crucial for improving sensitivity in the immunoassay systems. The majority of enzymes or nanoparticles as high-sensitivity probes are very popular in immunoassays^1–5^. To greatly improve the sensitivity and specificity of protein detection, nucleic acids amplification technique was employed to quantify protein, Le group has reported an affinity aptamer polymerase chain reaction (PCR) technique for ultrasensitive detection of proteins^6^. Meanwhile, immunopolymerase chain reaction (immuno-PCR)^7–9^ and immuno-rolling circle amplification (immuno- RCA)^10, 11^ approaches have also been developed to profile protein. Although these techniques offer very high detection sensitivity, they require some special requirements such as the ligation of a padlock probe, a high precision thermal cycler, or complicated procedures, preventing these approaches from being widely used. Thus, there is a pressing need for new analytical technologies capable of the efficient analysis of low-abundance proteins. Immuno-LAMP is a well-known technique for the sensitive detection of proteins. Pourhassan-Moghaddam *et al*. reported “Protein detection through different platforms of immuno-loop-mediated isothermal amplification”^12^. In the case of aptamer, it is possible to directly use it as the substrate for the Immuno-LAMP. Sometimes further amplification of the signal may be necessary, particularly in the case of detecting trace proteins, such as application of nanoprobes, integration with signal DNA-containing liposome. In previous work, our research group has developed an immuno-loop-mediated isothermal amplification for ultrasensitive detection of mucin 1 biomarker with target aptamer used as primer F3 in LAMP^13^. In order to reduce background interference and overcome known target aptamers restriction, in this work, a dual amplification strategy of using immuno-LAMP based on DNA-encapsulating liposomes was proposed for the determination of low-abundance multiple proteins. Recently, LAMP has attracted great interest, because of its unique advantages of under isothermal conditions (60–65 °C), high specificity and sensitivity using a set of six specially designed primers and a BstDNA polymeras within 2 hours^14–16^. Due to such advantages, LAMP has become a powerful tool for nucleic acid amplification and it has already been applied widely in pathogen detection^17^, tumor detection^18^, organophosphorus pesticides^19^ and embryo sex identification^20^. However, to the best of our knowledge, no investigator has reported on magnetic immuno-LAMP assay for trace amounts of protein analysis based on DNA-encapsulating liposomes.

Liposomes are spherical phospholipid bilayer vesicles^21^ which consist of an aqueous inner cavity delimited by a cell-membrane-like phospholipid bilayer presenting, on each side, the polar heads of the amphiphile. Because of their unique chemical and physical properties as well as their three-dimensional structure, they have become one of the most popular nanocarriers in the fields of nanodevices, drug delivery^22^ and gene delivery^23^ as well as a mimic for cell membranes^24^. Furthermore, in recent decades, liposomes have attracted great attention as a mean to amplify the signal in the bioanalysis field. Liposomes can encapsulate various signal substances, such as oligonucleotides^25^, colorimetric dyes^26^, fluorophores^27^, electrochemical markers and chemiluminescent molecular^28^. In these assay strategies, the signal amplification can be obtained by detecting large amounts of signal marker molecules which can be released from liposomes. The unique amphiphilic structures of liposomes make them prominent cargo carriers to engulf many DNA target as the signal molecule.

Moreover, we utilized P-glycoprotein (P-gp) as a model protein, a 170,000 daltons trans-membrane protein^29, 30^ which is present in many multidrug- resistance (MDR) cell lines that is thought to act as an energy-dependent drug efflux pump for reducing intracellular concentrations of chemotherapeutic agents^31^. In numerous tumors, the expression level of P-glycoprotein, and related members of the ATP-binding cassette transporter family multidrug-resistance associated protein (MRP) is a significant prognostic indicator at the time of diagnosis^32, 33^. Moreover, high levels of P-glycoprotein are discovered in a high proportion of relapsing cancers^34, 35^. Thus, to better elucidate P-gp functions, effective detection technologies for low levels of P-glycoprotein are urgently needed.

In this paper, by taking advantage of the merits of LAMP and liposomes, we first developed a DNA encapsulated liposome-LAMP amplification method for the ultrasensitive quantification of low abundance proteins (P-glycoprotein). In comparison with traditional ELISA, our method substantially improves the detection sensitivity and dynamic range. Furthermore, this magnetic liposome-Im-LAMP technique is not limited to the detection of P-gp, it can be applied to analysis of any other low-abundance proteins.

## Results and Discussions

### Fabrication of magnetic liposome-LAMP immunoassays

**Figure 1:**
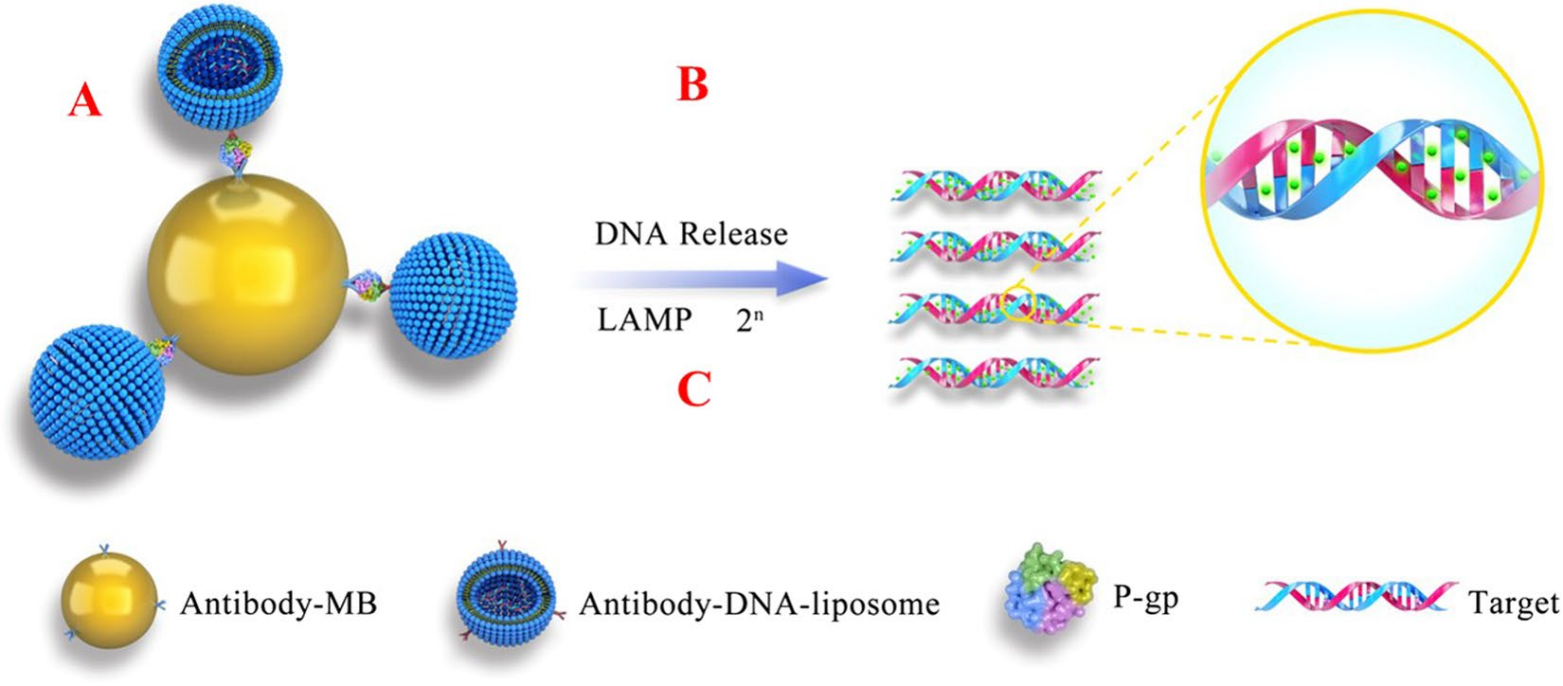
Schematic representation of magnetic liposome-Im-LAMP assay. (**A**) The presence of P-gp mediates the formation of a sandwiched immunocomplex between Antibody-tagged DNA-encapsulating liposomes and antibody-tagged magnetic beads; (**B**) liposomes are lysed with the surfactant to release the encapsulated DNA target; (**C**) DNA targets initiate LAMP reaction yielding fluorescence signal amplification.

For sensitive detection of P-gp, a typical sandwich format with the DNA-encapsulating immuno-liposomes as the detection reagent and magnetic microbeads coating with relavant antibodys were constructed. The design of magnetic liposome-LAMP immunoassay is illustrated in Fig. 1, the DNA-encapsulated liposomes-antibody complex contains hundreds of DNA target, and carboxyl-modified magnetic beads were covalently conjugated with the UIC2 antibody. The presence of P-gp leads to the generation of a sandwich hybrid which consists of the DNA-encapsulated liposome–antibody, a magnetic bead-modified antibody, and the target protein. The sandwich hybrids were separated from the free proteins by the magnetic field. Surfactant triton X-100 was subsequently used to disrupt the liposome to release a large number of DNAs. The DNA targets were designed to initiate the LAMP reaction in the presence of BstDNA polymerase and dNTPs. Eventually, each target DNA can undergo the LAMP reaction generating fluorescence signal, which can be employed as a quantitative measure for the P-gp concentration in the samples. Thus, dual signal amplification strategy (DNA-encapsulated liposomes and LAMP amplification) can give rise to a great increase of the fluorescence signal.

### Characterization of the DNA encapsulating liposome

**Figure 2:**
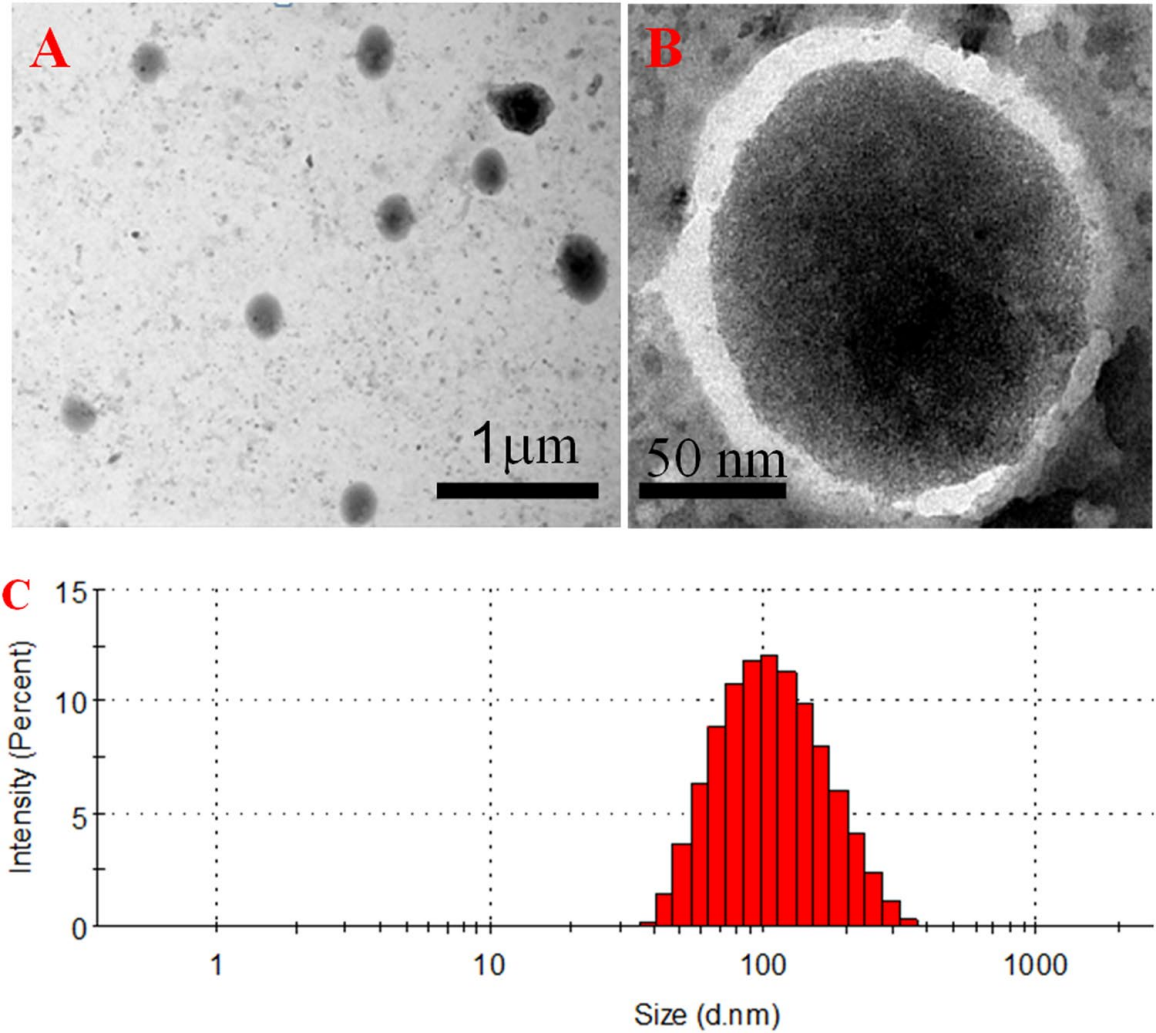
TEM imagines of DNA-encapsulating liposomes (**A** and **B** from lower magnification to higher magnification); The size distribution histogram of DNA-encapsulating liposomes (**C**).

In this work, the DNA target was encapsulated into liposomes as signal reporter via the LAMP reaction. The as-obtained DNA-encapsulated liposomes in this work were evaluated by transmission electron microscope (TEM) and dynamic light scattering (DLS). The TEM images show that DNA-encapsulated liposomes were spherical in shape and the size of the liposome was approximately 150 nm (as shown in Fig. 2). Dynamic light scattering technique also demonstrated that the hydrodynamic size (diameter) of the DNA-encapsulated liposomes was 145 ± 2 nm, consistent with the TEM images (Fig. 2C). The contrast between the dark center and pale edge of the DNA-encapsulated liposomes with higher magnification provides direct proof for their core/shell structure. The pale shell is considered to be a lipid membrane, as phosphotungstic acid is incapable of staining it, while it can successfully stain DNA molecules. Furthermore, the TEM images visually indicated that the DNA target was successfully encapsulated inside liposomes.

### Analytical performance of magnetic liposome-LAMP immunoassay for P-gp detection

The concentration of the DNA template entrapped is a critical factor for LAMP immunoassays. Therefore, the influence of the concentration of the DNA target on the assay performance was investigated in this work. Concentrations of template DNA-encapsulated (10^7^ copies/µL, 10^6^ copies/µL, 10^5^ copies/µL, and 10^4^ copies/µL) liposome was used for LAMP assay. The results suggest that the best signal amplification could be obtained with a concentration of 10^7^ copies/µL. Thus, an optimal concentration of DNA-entrapped was selected for the subsequent assays. In order to achieve the best performance, the effects of release agents on the assay performance were also investigated. Release agents such as methanol, ethanol, n-propyl alcohol, Triton X-100 were commonly used to lyse the liposomes. In order to improve the release efficiency of the DNA template from the liposome, releasing agents mentioned above were chosen and optimized. The result indicated that 10 mM Triton X-100 was suitable for the LAMP amplification compared with other agents. In addition, we observed that effect on the efficiency of the LAMP reaction. Fortunately, result shows that the presence of 10 mM Triton X-100 has little influence on the efficiency of LAMP reaction. Thus, 10 mM Triton X-100 is applied as liposome lysis reagent in the liposome-LAMP immunoassay.

**Figure 3:**
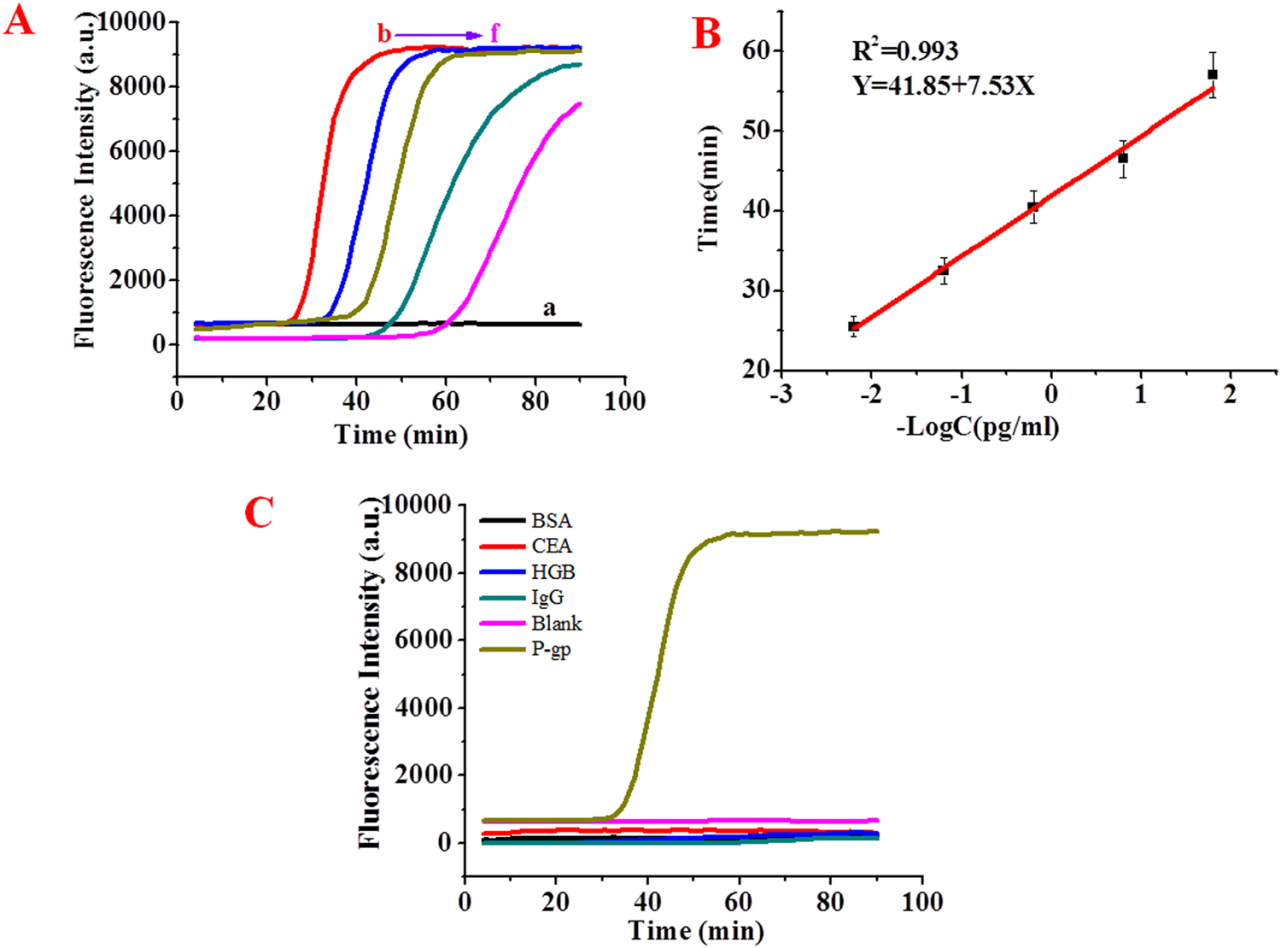
The real-time fluorescence curves of magnetic liposome-Im-LAMP assay reactions with different concentrations of P-gp:0 pg/ml (curve a), 160 pg/ml (curve b), 16 pg/ml (curve c), 1.6 pg/ml (curve d), 0.16 pg/ml (curve e), 1.6*10^−2^ pg/ml (curve f). (**B**) The relationship between cycle time and log concentrations of P-gp. The results are average values of three repetitive measurements. (**C**) Specificity of magnetic liposome-Im-LAMP assay detection of P-gp: the real-time fluorescence curves of Im-LAMP reactions with different reference proteins, bovine serum albumin (BSA), carcinoembryonic antigen (CEA), hemoglobin (HGB), immunoglobulin G (IgG) and the blank. The Im-LAMP reactions were performed in a volume of 10 µL. [FIP] = [BIP] = 1.6 µM, [B3] = [F3] = 0.2 µM, [Bst DNA polymerase] = 8 U/µL.

Figure 3A depicts the real-time fluorescence curves of Im-LAMP reactions with different concentrations of P-gp. It is observed that the cycle time (Ct value, defined as the reaction time necessary for samples to attain sufficiently positive response above the baseline during real-time fluorescence amplification) decreased with increasing P-gp concentration in the range from 0.016 pg mL^−1^ to 160 pg mL^−1^. Furthermore, Fig. 3B depicts the calibration curve of the liposome-LAMP immunoassays, Ct value is linearly dependent on the logarithm (log) of the concentration of P-gp ranging from 0.016 pg mL^−1^ to 160 pg mL^−1^. The correlation equation is Y = 41.85–7.53 Log(CP-gp) (Y: Ct; C: the concentration of P-gp, pg/mL), and the corresponding correlation coefficient R is 0.993, which shows a good correlation between the concentration of the P-gp and the Ct values. The detection limit as low as 0.005 pg mL^−1^ was estimated in terms of the 3 times the standard deviation. Such a detection limit (LOD) for P-gp was 3–4 orders of magnitude lower than reported by sensitive ELISA for P-gp detection. The dynamic range was about 2–3 greater orders of magnitude than ELISA methods (Table 1). In addition, this platform has substantially reduced sample consumption. Thus, the developed liposome-LAMP immunoassay indicates a high sensitivity for P-gp determination.

**Table 1:** Comparison of magnetic liposome-Im-LAMP detection profiling for P-gp with conventional ELISA approach.

Approach	Linear range (pg/mL)	Detection limit (pg/mL)	Sample volume (µL)
ELISA	10 to 320	5	50
Magnetic liposome-Im-LAMP	1.6*10^−2^ to 160	5*10^−3^	10

### The specificity of the magnetic liposome-Im-LAMP assay

To evaluate the specificity of the proposed magnetic liposome-Im-LAMP immunoassay, other proteins such as bovine serum albumin (BSA), carcinoembryonic antigen (CEA), hemoglobin (HGB), immunoglobulin G (IgG) were selected as reference proteins (100 pg/mL). The real-time fluorescence responses of the developed magnetic liposome-Im-LAMP immunoassay to these proteins are shown in Fig. 3C. It was observed that, only P-gp generated the LAMP reaction, other proteins do not initiate fluorescence amplification. Therefore, the proposed magnetic liposome-LAMP immunoassay exhibits high specificity for the P-gp detection.

## Conclusions

In summary, this novel magnetic liposome-Im-LAMP immunoassay for the ultrasensitive detection of protein was developed by combining the Loop-mediated isothermal amplification with magnetic immunoassay. This technique provided a dual-signal amplification strategy for P-gp detection with remarkably enhanced sensitivity and specificity, attributed to primary amplification via releasing a large number of DNA targets from the liposome, followed by a secondary LAMP amplification. Antibody modified micro-magnetic-beads were incorporated into the technique to identify the P-gp and exert magnetic separation. Although the P-gp system is used for the proof-of-concept, the approach should be general for almost any targets with known relevant antibodies. Moreover, it was demonstrated that this method has significant advantages of high sensitivity and specificity, and low sample consumption. Therefore, our proposed dual-signal amplification strategy holds a great potential for multiple low-abundance proteins detection in proteomics and clinical diagnostics.

## Methods

### Reagents and Apparatus

Glutaraldehyde (GA) solution (25%), Tween 20, chloroform and methanol were purchased from the Sinopharm Chemical Reagent Co., Ltd. (China). P-Glycoprotein Monoclonal Antibody (C219) and P-Glycoprotein Monoclonal Antibody (UIC2) were purchased from ThermoFisher Scientific, N-Hydroxysuccinimide sodium salt (NHS, 98%) was obtained from Aladdin Industrial Corporation (Shanghai), 1-ethyl-3-(3-dimethylaminopropyl) carbodiimide hydrochloride (EDC, C_8_H_17_N_3_HCl), carcinoembryonic antigen (CEA), hemoglobin (HGB), immunoglobulin G (IgG) and Bovine serum albumin (BSA), Cholesterol, 1, 2-dipalmitoyl-*sn*-glycerol-3-phos phatidylethanolamine (DPPE), and 1,2-dipalmitoyl-*sn*-glycero-3-phosphor -choline (DPPC) were purchased from Sigma-Aldrich Chemical Co (USA). LAMP amplification reagent (DNA Amplification kit) was purchased from Deaou Ltd. (Guangzhou, People’s Republic of China). The DNA sequences: FIP, BIP, B3, F3, and the template DNA (Table S1 in supporting information) used in this study were synthesized and purified with high-performance liquid chromatography (HPLC) by Sangon Biotechnology Co. Ltd. (Shanghai, People’s Republic of China). The process of the LAMP amplification was monitored by a real-time fluorescence PCR system (BIOER LineGene 9640). The morphologies of the liposomes were investigated using transmission electron microscopy (TEM; JEOL 2011). The hydrodynamic size of liposomes was measured by dynamic light scattering with a Malvern ZS90 particle size analyzer (Malvern Instruments, UK).

### Primer design

Appropriate design of the primers is crucial for gene amplification using the LAMP method. The specific LAMP primers based on the nucleotide sequence of template DNA were designed by LAMP primer designing software (Primer Explorer V4), an online primer designing tool developed by Eiken Chemical Co. LTD, Japan (http://primerexplorer.jp/e/).

### Preparation of DNA-Encapsulating Liposomes

DNA encapsulating liposomes were prepared using the film hydration method according to the procedure from the literature with slight modifications^36^. In brief, DPPC, cholesterol, and DPPE (18.5 mg, 1.75 mg, 9.75 mg) were dissolved in 4 mL of chloroform/methanol (3.4 mL and 0.6 mL, respectively), followed by sonication treatment to ensure homogeneous mixing. After sonicating for 10 min, the mixture was then placed onto the rotary evaporator, and the organic solvent was removed by rotary evaporation under reduced pressure (0.09 MPa) at 36 °C to form a thin lipid film on the inside wall of the round-bottom flask. Afterward, the film was hydrated in 2 mL of 0.1 M PBS containing a different concentration of DNA target at 46 °C with the vigorous shaking until the lipid film was peeled off the inside wall. Finally, to achieve unilamellar vesicles, the multilamellar liposomes were applied for a sonication treatment (15 min of 10 s on and 10 s off) in an ice-water bath by a probe-type sonicator. In order to remove undispersed lipids and multilamellar vesicles, the resulting liposome suspension was centrifuged at 4000 rpm for 15 min. The unencapsulated DNA oligonucleotides were eliminated from the liposome suspension through ultrafiltration centrifugation treatment using the ultrafiltration tube with a certain molecular weight cutoff. The obtained DNA encapsulating liposome suspension was stored at 4 °C for future use.

### Conjugation of Antibodies to Liposomes

Liposome conjugates with P-glycoprotein monoclonal antibody (C219) which may react with an internal epitope of P-gp were prepared via glutaraldehyde coupling method according to literatures with slight modifications^37, 38^. In brief, in 3 mL of 2.5% glutaraldehyde solution, 2 mL of DNA-encapsulating liposomes (4 mg lipid mL^−1^) was added in drops under gent stirring for 1 h at 25 °C. Excess glutaraldehyde was deposed by ultrafiltration centrifugation 3 times. Then, 1 mL of P-glycoprotein (human) monoclonal antibody (1 mg mL^−1^ in PBS) was added under gentle stirring following the incubation for 2 h at 37 °C. In order to block excess aldehyde groups on the liposome surface, 1 mL of BSA (10 mg mL^−1^ in PBS) was added and reacted under gentle stirring for 1 h at 25 °C. The uncoupled BSA and P-gp monoclonal antibody were removed from the liposomes by ultrafiltration centrifugation at 100,000 rpm for 15 min. The resulting DNA encapsulating liposomes were stored in the 10 mM phosphate buffer (pH 7.2) containing 3% (w/w) BSA at 4 °C until use.

### Preparation of Antibody Modified Microbeads

The magnetic beads modified with the P-gp monoclonal antibody were prepared by using the EDC-NHS activation, as briefly described as follows: The carboxyl-coupled microbeads (10 mg/ml) were first washed using 2 mL of 2-(N-Morpholino) ethane sulfonic acid buffer (MEST, pH 6.0, 0.05% Tween) three times with the aid of centrifugation and resuspended in MEST with a final concentration of 5 mg mL^−1^. Then 0.2 M NHS (23 μL) and 0.2 M EDC (26 μL) were added to magnetic beads solution (5 mg/ml) to activate the carboxy group of magnetic beads. After activation, the supernatant was removed by magnetic separation and washed by MEST three times, and finally resuspended in 500 μL PBS (10 mM, pH 7.4). In a 500 μL of activated carboxyl-coupled microbead solution, 100 μL of P-Glycoprotein Monoclonal Antibody (UIC2) was added and incubated for 3 h at 37 °C. In order to eliminate nonspecific binding of magnetic beads, the P-gp-modified microbeads were blocked with 1 ml PBST (pH 7.4, 1% BSA) for 30 min and washed using 2 mL of PBS three times with the aid of magnetic separation. The sediment was collected and resuspended in 0.2 mL of PBS, and the resulting P-gp-modified microbeads solution was stored at 4 °C for future use.

### DNA Encapsulating Liposome-LAMP immunoassay

50 μL of P-gp sample (concentration ranging from 0 to 160 pg mL^−1^) in PBS (pH 7.2) containing 3% (w/w) BSA was mixed with 40 μL P-gp-modified microbeads and incubated for 1 h at 37 °C. The unbound sample solution was discarded by centrifugation and magnetic separation, and the sediments were washed using PBS (pH 7.2) containing 3% (w/w) BSA three times. Then, 10 μL of DNA-encapsulating immunoliposomes (4 mg lipid mL^−1^) were added into the above solution and incubated for 1 h at 37 °C. The uncombined liposome mixture was removed, washed using PBS containing 3% (w/w) BSA three times. Subsequently, in order to lyse the bound liposomes, 30 μL of Triton X-100 (10 mM) was added into above mixture, incubated for 30 min at 37 °C. Lysates were collected and transferred into a microcentrifuge tube for further use. The DNA target released from the liposomes could initiate LAMP reaction, as described as follows: 10 μL of lysate containing DNA targets were mixed with Tris-HCl (pH 8.8), 10 mM KCl, 8 mM MgSO_4_, 10 mM (NH4)_2_SO_4_, 0.1% Tween 20, 0.8 M Betaine, 25 µM Calcein, 0.5 mM MnCl_2_, 1.4 mM dNTPs, 8 U Bst Polymerase, 0.2 µM of the outer primer (B_3_), and 1.6 µM each of the inner primer (FIP/BIP), subsequently, the mixture was heated to 63 °C for 90 mins.

Calcein is an important metal indicator that yields a strong fluorescence signal by forming complexes with divalent metallic ions, which is used for various analyses. The real-time process of the LAMP reaction was monitored by a real-time fluorescence PCR system.

### Data availability statements

The datasets generated during and/or analysed during the current study are available from the corresponding author on reasonable request.

## Electronic supplementary material

Supplementary Information
