# Supplementary material for: Magnetic-Immuno-Loop-Mediated Isothermal Amplification Based on DNA Encapsulating Liposome for the Ultrasensitive Detection of P-glycoprotein
Source: Sci Rep. 2017 Aug 24;7:9312. doi: 10.1038/s41598-017-10133-3 (PMC5571029; doi:10.1038/s41598-017-10133-3)
Supplement: Supplementary file 1 — Supplementary Information [file 41598_2017_10133_MOESM1_ESM.doc]

**Supporting Information**

**Magnetic-Immuno-Loop-Mediated Isothermal Amplification Based on DNA Encapsulating Liposome for the Ultrasensitive Detection of P-glycoprotein**

**Hongmei Cao^a^, Xueen Fang^a*^, Peng Liu^b^, Hua Li^a^, Weiwei Chen^a^, Baohong Liu^a^ and Jilie Kong^a*^**

*^a^Department of Chemistry and Institute of Biomedi**cal Sciences, Fudan University,Shanghai 200433, PR China*

*^b^Department of Laboratory Diagnosis, Changhai Hospital, Second Military Medical University, 168 Changhai road, Shanghai, 200433, China*

Table S1. The sequence of primer (F3, B3, FIP and BIP) and DNA target

template.

| F3 | TTGTTCCTGCTCCACTCT |
| --- | --- |
| B3 | TTCTTCATTGATCTCCTGTAGC |
| FIP | AGCTAGAGCTTGATGGGGATCACGTGGTTATCAAGCTCCAA |
| BIP | GTTATCAAGCTTCTGCAGGTCCTCTGACCTTGTTGAGGCTTT |
| Target | TTGTTCCTGCTCCACTCTTCCTTTTGGGCTTTTTTGTTTCCCGCTCTAGCGCTTCAATCGTGGTTATCAAGCTCCAAACACTGATAGTTTAAACTGAAGGCGGGAAACGACAATCTGATCCCCATCAAGCTCTAGCTAGAGCGGCCGCGTTATCAAGCTTCTGCAGGTCCTGCTCGAGTGGAAGCTAATTCTCAGTCCAAAGCCTCAACAAGGTCAGGGTACAGAGTCTCCAAACCATTAGCCAAAAGCTACAGGAGATCAATGAAGAATCTTCAATCAAAGTAAA |
